# Online advertising and marketing claims by providers of proton beam therapy: are they guideline-based?

**DOI:** 10.1186/s13014-018-0988-z

**Published:** 2018-03-15

**Authors:** Mark T. Corkum, Wei Liu, David A. Palma, Glenn S. Bauman, Robert E. Dinniwell, Andrew Warner, Mark V. Mishra, Alexander V. Louie

**Affiliations:** 1 Department of Radiation Oncology, London Health Sciences Centre, 790 Commissioners Road East, London, ON N6A 4L6 Canada; 2 Department of Epidemiology and Biostatistics, Western University, London, Canada; 3 Departments of Radiation Oncology, University of Maryland School of Medicine, Baltimore, USA

## Abstract

**Background:**

Cancer patients frequently search the Internet for treatment options, and hospital websites are seen as reliable sources of knowledge. Guidelines support the use of proton radiotherapy in specific disease sites or on clinical trials. This study aims to evaluate direct-to-consumer advertising content and claims made by proton therapy centre (PTC) websites worldwide.

**Methods:**

Operational PTC websites in English were identified through the Particle Therapy Co-Operative Group website. Data abstraction of website content was performed independently by two investigators. Eight international guidelines were consulted to determine guideline-based indications for proton radiotherapy. Univariate and multivariate logistic regression models were used to determine the characteristics of PTC websites that indicated proton radiotherapy offered greater disease control or cure rates.

**Results:**

Forty-eight PTCs with 46 English websites were identified. 60·9% of PTC websites claimed proton therapy provided improved disease control or cure. U.S. websites listed more indications than international websites (15·5 ± 5·4 vs. 10·4 ± 5·8, *p* = 0·004). The most common disease sites advertised were prostate (87·0%), head and neck (87·0%) and pediatrics (82·6%), all of which were indicated in least one international guideline. Several disease sites advertised were not present in any consensus guidelines, including pancreatobiliary (52·2%), breast (50·0%), and esophageal (43·5%) cancers. Multivariate analysis found increasing number of disease sites and claiming their centre was a local or regional leader in proton radiotherapy was associated with indicating proton radiotherapy offers greater disease control or cure.

**Conclusions:**

Information from PTC websites often differs from recommendations found in international consensus guidelines. As online marketing information may have significant influence on patient decision-making, alignment of such information with accepted guidelines and consensus opinion should be adopted by PTC providers.

**Electronic supplementary material:**

The online version of this article (10.1186/s13014-018-0988-z) contains supplementary material, which is available to authorized users.

## Background

The internet is a widely accessed source of medical information for patients [1–8]. Direct-to-consumer advertising (DTCA) on hospital and healthcare organization websites is common [9], however frequently do not adequately support their claims with reference to available evidence [10]. While print and broadcast media must conform to nationally regulated standards, there is currently no regulation of DTCA by healthcare websites despite potential conflict of interests [3, 10, 11].

Controversial DTCA for new oncology treatments has been previously identified [9, 12]. Patients with cancer may be especially vulnerable to controversial marketing due to associated fear and anxiety [11, 13]. As cancer patients may place more confidence in online information when endorsed by professional bodies and organizations [3, 9], it has been argued that strict guidelines and more oversight should be implemented for marketing by cancer centres to patients with cancer [9–11, 13].

Proton beam therapy (PBT) is a rapidly emerging technology with desirable dosimetric advantages compared to traditional photon radiotherapy (RT). PBT takes advantage of the “Bragg peak”, allowing for deposition of radiation to a tumour target with minimal to no exit dose affecting normal tissues beyond. Routine adoption of proton beam therapy faces several challenges. High capital and operating costs create barriers to institutional and individual patient access to PBT [14], particularly as insurers may be reluctant to cover treatments where there is an insufficient evidence base to justify higher cost PBT compared to lower cost alternatives [15, 16]. Despite these barriers, utilization of PBT is increasing [17], mirroring the rapid rise in number of PBT centres worldwide. Controversial online advertising practices with PBT have previously been identified, targeted towards men with prostate cancer [18]. International published guidelines only recommend PBT for a limited number of disease sites [19–27].

While heavy ion radiotherapy dates as far back as the 1930’s, it was only after Loma Linda opened a three-gantry facility in 1990 that the number of PBT centres began to rise rapidly [28]. As of September 2016, there were 62 operating PBT internationally, including 25 in the United States, 11 in Japan, and 6 in Germany [29]. There are 37 additional PBT centres under construction around the world [30]. Business models for PBT differ across these jurisdictions and ultimately, regardless of payer, such facilities become economically viable only when there are sufficient patient volumes to fully utilize such facilities. The purpose of our study was to compare DTCA content and claims on proton therapy centre (PTC) websites against current evidence based indications (as reflected in published guidelines) to characterize the frequency and nature by which PBT is being promoted for non-evidence based indications.

## Methods

### Study population

All operating PTCs worldwide as of September 1, 2016 were identified on the Particle Therapy Co-operative Group website (https://www.ptcog.ch/). The website of each centre was reviewed to confirm its operational status. We excluded PTCs without an English website and three centres that solely treat ocular tumours, as they lack sufficient energy to treat deeper tumours. The websites of facilities under construction and facilities in planning stage identified by the Particle Therapy Co-operative Group were then reviewed and one additional operational centre was identified. A separate Internet search did not reveal any additional centres. We also searched the websites of the five PBT manufacturers with centres in the United States to confirm that our list of PTCs was complete. Our final study population consisted of forty-eight operational PTCs and forty-six PTC websites.

### Data collection

The websites of PTCs were evaluated using a standardized data collection form, adapted from previously published studies assessing hospital website DTCA [9, 12]. The data collection form is available in Additional file 1: Appendix A. In two instances, two PTC’s were represented by a single website; data was collected once for these PTCs. Data was collected independently by two investigators (MC and WL), with discrepancies reached by consensus after further review of the websites and discussion with a third investigator (AVL).

We evaluated for advertising prominence and compliance with the American Medical Association Opinion on Advertising and Publicity [31, 32]. Data collected from each centre’s website included: disease sites that were claimed to be treatable with PBT, whether PBT was available as a clinical trial and details regarding accessibility (cost, travel). Data regarding claims of efficacy, decreased morbidity, and/or comparisons between PBT and RT were also collected. Website traffic was evaluated using Alexa Rank (http://www.alexa.com) [33]. Population estimates for the region served by the PBT centre were obtained from Population Explorer (https://populationexplorer.com), an international tool developed by Kimetrica for the U.S. Agency for International Development’s Famine Early Warning System Network. Academic practices were defined as those associated with a medical school and/or residency training program; where doubt existed, supplemental internet searches were performed to clarify.

Eight international guidelines were consulted to determine disease sites potentially suitable for treatment with proton radiotherapy [19–27]. According to these guidelines, disease sites that were definitely or potentially indicated for PBT by two or more guidelines included: pediatric, eye/orbit, benign central nervous system (CNS), low-grade glioma, other CNS (e.g. spine/bone, base of skull), liver, head and neck, lymphoma, sarcomas and recurrent disease. Four disease sites (palliative treatments, prostate, lung and bone cancers) were only mentioned by a single guideline, and were included in our study as indicated sites. If a website listed more generic terms (e.g. glioma instead of low-grade glioma, base of skull or spine tumour instead of chordoma/chondrosarcoma, etc.), it was included as a potentially indicated PBT despite the possibility this could include non-indicated diagnoses. All other disease sites collected were considered not to be endorsed by the consensus international guidelines for PBT treatment.

### Statistical analysis

Descriptive statistics were generated for all websites and compared between those advertised in the U.S. versus internationally using the chi-square test, Fisher’s exact test, two-sample T-test or Wilcoxon rank sum test as appropriate. Where more than one PTC was represented by a single website, attempts were made to collect separate demographic data and report it separately. For variables analyzed at a website level, data were not counted multiple times. This study was written to meet requirements published within the STROBE statement [34].

Univariate and multivariate logistic regression was performed to identify characteristics of websites that were predictive of greater disease control or cure rates. Explanatory variables included in the univariate logistic regression analyses were determined a priori. Multivariate logistic regression initially included univariate variables with a *p*-value < 0.10 for each end point. These variables were sequentially removed using backward elimination until all remaining covariates had *p*-values < 0.05. To allow multivariate analysis for websites representing two centres, we chose to include demographic data from the PTC with the greatest surrounding population. All statistical analysis was performed using SAS version 9.4 software (SAS institute, Cary NC), using two-sided statistical testing at the 0.05 significance level.

## Results

Forty-eight PTC centres were identified resulting in forty-six PTC websites for analysis. Additional file 2: Appendix B summarizes included and excluded PTC’s including reason for exclusion. Features of included websites are presented in Table 1. Twenty-four PTCs (with twenty-two websites) were from the United States, 62.5% were considered academic centres and 47.8% of webpages listed the manufacturer of their proton treatment machine, of which 45.5% claimed that their machine was superior. PBT-specific patient testimonials were present on 43.5% of websites, and 63.0% included a section dedicated to out-of-town patients. The amount of information contained within each website varied greatly, from single webpages only containing general information to detailed discussions about the merits of PBT for specific disease sites.

**Table 1:** Information about Proton Therapy Centre Websites

	All Websites % (*n*) (*N* = 46 unless otherwise specified)
Proton Centre Location (*N* = 48)	
United States	50.0% (24)
Asia	31.3% (15)
Europe	16.7% (8)
Africa	2.1% (1)
Website from an Academic Proton Centre (*N* = 48)	62.5% (30)
Another Proton Centre within 300 km (*N* = 48)	52.1% (25)
Population Estimate within 100 km of Proton Centre (*N* = 48)	
≤ 2,499,999	18.8% (9)
2,500,000–4,999,999	25% (12)
5,000,000–9,999,999	20.8% (10)
≥ 10,000,000	35.4% (17)
Number of Clicks to First Page Mentioning Proton Therapy	
0 (directly on homepage)	56.5% (26)
1	26.1% (12)
≥ 2	17.4% (8)
Manufacturer of Machine Listed	47.8% (22)
Claim Their Proton Machine Superior (*N* = 22)	45.5% (10)
Stock Material from Manufacturer Present (*N* = 22)	31.8% (7)
Stock Material Acknowledged from Manufacturer (*N* = 7)	42.9% (3)
Link to Manufacturer Website Present (*N* = 22)	13.6% (3)
Number of Clicks to Mention of Manufacturer (*N* = 22)	
0 (directly on homepage)	13.6% (3)
1	40.9% (9)
≥ 2	45.5% (10)
Out-of-Town Patient Section on Website	63.0% (29)
Proton Therapy Patient Testimonials on Website	43.5% (20)

Selected treatable disease sites listed on PTC websites are presented in Table 2. A full list of disease sites mentioned on websites is available in Additional file 3: Appendix C. The mean number of disease sites listed on all websites was 12.8 ± 6.1, with 15.5 ± 5.4 for U.S. compared to 10.4 ± 5.8 for international (*p* = 0.004). The most common disease sites listed on websites were prostate (87.0%), head and neck (87.0%), and pediatric tumours (82.6%). Several disease sites advertised were not present in any international consensus guidelines, including pancreatobiliary (52.2%), breast (50.0%), esophageal (43.5%), colorectal (39.1%) and gynecologic (30.4%) cancers.

**Table 2:** Proportion of Selected Disease Sites Mentioned on Proton Therapy Centre Websites

Site	All Websites % (n)(*N* = 46)	US Websites % (n) (*N* = 22)	International Websites % (n) (*N* = 24)	US v. Int’l *p*-value
Number of Sites Listed				
(mean ± SD)	12.8 ± 6.1	15.5 ± 5.4	10.4 ± 5.8	***0.004***
Adult CNS (any)^*^	89.1% (41)	95.5% (21)	83.3% (20)	0.349
Benign	58.7% (27)	63.6% (14)	54.2% (13)	0.515
Glioma (High or Low Grade)	45.7% (21)	50.0% (11)	41.7% (10)	0.571
Other (e.g. base of skull)	65.2% (30)	59.1% (13)	70.8% (17)	0.404
Spine	58.7% (26)	77.3% (17)	41.7% (10)	***0.014***
Bladder	19.6% (9)	27.3% (6)	12.5% (3)	0.276
Breast	50.0% (23)	77.3% (17)	25.0% (6)	***< 0.001***
Eye/Orbit	58.7% (27)	68.2% (15)	50.0% (12)	0.211
GI (any)^*^	76.1% (35)	90.9% (20)	62.5% (15)	***0.024***
Anal	17.4% (8)	36.4% (8)	0% (0)	***0.001***
Colorectal	39.1% (18)	54.6% (12)	25.0% (6)	***0.040***
Esophagus	43.5% (20)	54.6% (12)	33.3% (8)	0.147
Liver	56.5% (26)	54.6% (12)	58.3% (14)	0.796
Pancreatobiliary	52.2% (24)	68.2% (15)	37.5% (9)	***0.037***
Stomach	17.4% (8)	31.8% (7)	4.2% (1)	***0.020***
Gynecologic	30.4% (14)	36.4% (8)	25.0% (6)	0.403
Head and Neck	87.0% (40)	90.9% (20)	83.3% (20)	0.667
Kidney	13.0% (6)	4.6% (1)	20.8% (5)	0.190
Lymphoma	41.3% (19)	68.2% (15)	16.7% (4)	***< 0.001***
Lung	76.1% (35)	95.5% (21)	58.3% (14)	***0.003***
Pediatric Tumours	82.6% (38)	95.5% (21)	70.8% (17)	***0.049***
Prostate	87.0% (40)	95.5% (21)	79.2% (19)	0.190
Recurrent Disease	54.4% (25)	54.6% (12)	54.2% (13)	0.979
Sarcoma	67.4% (31)	81.8% (18)	54.2% (13)	***0.046***

*These sites contain additional categories not presented, available in Appendix C

All entries with *p*<0.05 are bolded with italic underline

Several disease sites were more frequently mentioned on U.S. websites in comparison to international websites, including spinal (77.3% vs. 41.7%; *p* = 0.014), anal (36.4% vs. 0%; *p* = 0.001), colorectal (54.6% vs. 25.0%, *p* = 0.040), stomach (31.8% vs. 4.2%; *p* = 0.020), breast (77.3% vs. 25.0%; *p* <  0.001), lymphoma (68.2% vs. 16.7%; *p* <  0.001), lung (95.5% vs. 58.3%; *p* = 0.003) and pediatric tumours (95.5% vs. 70.8%, *p* = 0.049).

Claims made by PTC websites are presented in Table 3. Sixty-one percent of websites claimed proton therapy provided improved disease control or cure, with 13.0% of websites stating that for at least one disease site PBT was the standard of care treatment. Sixty-one percent of websites stated that proton therapy was more effective (i.e. improved survival or local control) than photon RT. Ninety-eight percent of websites stated there was a dose distribution advantage to proton therapy: 84.8% stated fewer side effects, 39.1% stated improved quality-of-life and 84.8% and 21.7% of websites stated decreased morbidity compared to photon RT and surgery respectively. Discussion about the cost/insurance coverage of proton therapy and availability in clinical trial setting was present in 69.6% and 56.5% respectively.

**Table 3:** Claims Made on Proton Therapy Centre Websites

	All Websites % (n) (*N* = 46 unless otherwise specified)
Claims about Proton Therapy	
Improved Disease Control or Cure	60.9% (28)
Standard of Care	13.0% (6)
Fewer Side Effects	84.8% (39)
Specific Side Effects Listed	67.4% (31)
Quicker Recovery Time	15.2% (7)
Increased Quality-of-Life	39.1% (18)
Dose Distribution Advantage	97.8% (45)
Claims and comparison to Other Modalities	
Photon RT: Increased Efficacy	60.9% (28)
Photon RT: Decreased Morbidity	84.8% (39)
Surgery: Increased Efficacy	2.2% (1)
Surgery: Decreased Morbidity	21.7% (10)
Evidence Provided for Claims on Website	
All Claims Referenced	4.4% (2)
Some Claims Referenced	32.6% (15)
Generic References (e.g. Studies show that…)	6.5% (3)
No Claims Referenced	56.5% (26)
Alternative Treatment(s) to Proton Therapy Listed	89.1% (41)
Claim Local/Regional Leader in Proton Therapy	43.5% (20)
Length of Time Treating/Patient Volume Listed	71.7% (33)
Mention of Cost/Insurance of Proton Therapy	69.6% (32)
Proton Therapy as Clinical Trial Listed	56.5% (26)

References were infrequently provided on websites: 4.4% provided references for all claims with 32.6% further websites providing a scientific reference for at least one claim. Seven percent stated that their claims were backed by scientific studies without a reference whereas 56.5% did not provide any references.

On univariate analysis, increasing number of disease sites listed (odds ratio (OR) per additional site: 1.14, 95% confidence interval (CI): 1.02–1.29, *p* = 0.025) and claiming to be a local/regional leader in PBT (OR: 4.67, 95% CI: 1.22–17.82, *p* = 0.024) were predictive of claiming better disease control or cure. Both of these factors remained significant on multivariate analysis (OR: 1.15, 95% CI: 1.02–1.31, *p* = 0.027 and OR: 5.15, 95% CI: 1.22–21.80, *p* = 0.026 respectively).

## Discussion

Proton radiotherapy is a rapidly expanding treatment modality that has attractive dosimetric advantages compared to photon radiotherapy, albeit at an increased capital and per-fraction reimbursement costs [14]. The main findings of this study are that worldwide, PTC websites commonly contain DTCA for PBT that are frequently not supported by evidence, and often advertise the treatment of cancer sites not currently endorsed by international consensus guidelines. This did not appear to be localized to a single centre, country or disease site; rather, this trend exists globally and for a multitude of cancer sites.

The ethical implications of DTCA within healthcare are neither new, nor unique to radiation oncology. Currently, the United States and New Zealand are the only two countries that permit print and broadcast DTCA within healthcare [35]; no countries regulate DTCA from online sources [11]. In the U.S., the Food and Drug Administration (FDA) regulates print and broadcast advertising: although only major risk information must be disclosed, they must direct consumers to other accessible sources of information on literature and all other associated risks [36]. Supporters of DTCA argue that it increases awareness, education and empowerment to facilitate patient-centred decision-making and decrease paternalistic care [37]. In contrast, arguments against DTCA include the high literacy level required, bypassing the patient-provider relationship, overutilization, and increased healthcare costs [37].

Cancer centres producing DTCA for new radiotherapy technologies was previously questioned with the introduction of Intensity Modulated Radiation Therapy (IMRT) to mainstream radiation oncology practices in the 2000’s [38]. While IMRT implementation was also questioned due to concerns of tumour targeting and risk of secondary malignancies (with larger volumes of normal tissues receiving low dose radiotherapy), the increased costs of IMRT were also a valid concern [39]. While the safety and accuracy of PBT have not been questioned to the same degree, the increased costs of PBT surpass those of IMRT, arising from both high capital costs plus increased reimbursement per fraction [14]. Traditional multi-room proton facilities cost in excess of 100 million USD [40], though single-room proton machines have now entered the market with a lower capital cost [41, 42]. Recent systematic reviews of the cost-effectiveness of PBT suggest superiority in several disease sites, such as particular pediatric brain tumours, left sided breast cancers at high risk of cardiac toxicity, selected head and neck cancers, and locally advanced lung cancers, acknowledging this finding is limited by the lack of published literature [43, 44].

As cancer care is highly profitable [45], it should not be surprising that spending on advertising is increasing. In 2014, $173 million was spent on cancer centre advertising in the U.S., roughly triple the amount compared to 2005 [46]. Of the 1500 cancer centres in the U.S., 20 accounted for 86% of advertising spending, of which 9 were NCI-designated [46]. Of these 20 centres, five were included in our study. The advertising practices of cancer centres have previously been brought into question, including appeals to the emotions of a vulnerable patient population, hope for survival, and improved outcomes without supporting factual information, while not mentioning costs or risks associated with treatment [11, 13, 46–48]. Our study supports these findings, demonstrating PTC websites frequently offer PBT to non-guideline recommended cancer sites, contain language which evokes hope for better treatment outcomes, and infrequently support all claims made on their website.

It has been argued that randomized clinical trials for PBT are not necessary due to the clear dose distribution advantages over RT [44, 49]. In addition, it has been argued that the capacity constraints of PBT centres precludes the ability to perform randomized clinical trials [50]. While some side effects are likely to be improved with PBT, it is possible that certain side effects, such as skin toxicity, could be expected to be worse with intensity modulated proton therapy [51]. There are over 100 clinical trials underway examining proton radiotherapy, albeit with only eight randomized clinical trials directly comparing proton versus photon radiotherapy [52]. Recently, the first presentation of a randomized study between proton and photon radiotherapy of 147 patients with locally advanced non-small cell lung cancer was published in abstract form, finding no difference in treatment failure or ≥ grade 3 radiation pneumonitis between the two treatment modalities [53].

In the absence of randomized data, we examined 8 guidelines on proton therapy to identify disease sites indicated for PBT [19–27]. These guidelines are from the U.S., Canada, Australia and New Zealand, the U.K., Denmark and the Netherlands. The publication dates range from 2009 to 2017. Guidelines differ on disease site indications, therefore when determining disease sites indicated by guidelines, we used a threshold of at least one guideline indicating a disease site. After review of the international guidelines, there were four disease sites only endorsed by a single guideline (NCCN guidelines). These four sites (prostate cancer, lung cancer, bone cancers and palliative tumours), were considered to be indicated sites for purposes of our study. Prostate cancer was considered an indicated site despite the NCCN guideline also clearly stating that “*They believe no clear evidence supports a benefit or decrement to proton therapy over IMRT for either treatment efficacy or long-term toxicity*” [54]. Despite retrospective and prospective cohort studies demonstrating favourable side effect profiles for PBT in several disease sites not mentioned in these guidelines (such as pancreas [55], breast [56], esophagus [57] and cervical [58] cancers), these were not considered indications in our study as they have not been cited in guidelines to date, and are subject to bias.

To our knowledge, this is the first systematic evaluation of DTCA on English PTC websites. However, our data is limited by inconsistencies between how disease sites were listed in international guidelines and websites. For example, many websites did not separate high and low grade gliomas. Several guidelines support the use of PBT for low grade gliomas [19, 21, 24], but no guidelines support the use of PBT in high grade gliomas. Other limitations of our study are excluding ten websites that were not in English. Several international websites that did have an English webpage contained limited information relative to equivalent U.S. websites. As such, our data for these websites may not be completely representative of DTCA towards the primary target audience. Inclusion of a disease site within a guideline is one measure of alignment with evidence based care but has limitations. Guidelines may be limited by the evidence base available or may be influenced by expert opinion depending on the methodology used to generate the guideline. We did not incorporate the availability of pencil beam scanning in our study. Some PTC’s offer both PBT and carbon ion therapy. At times, it was difficult to separate claims for PBT and carbon ion therapy. Finally, the nature of DTCA by an institution does not necessarily reflect the level of discussion that occurs at the provider/patient level. For example, while an indication might be listed on a DTCA website, an informed discussion with the patient may include discussions of alternatives and tailoring of recommendations to that individual’s situation. Such provisos that acknowledge treatments need to be individualized (“check with your doctor if drug x is right for you”) are a mainstay of other forms of DTCA but were not a prominent feature of the websites we examined.

## Conclusions

Our study shows that PTC websites contain DTCA for disease sites that are not endorsed by international consensus guidelines, in addition to appealing to the emotions of patients by often claiming superior disease control or decreased side effect profiles. This follows the pattern of other cancer treatments such as robotic surgery for prostate cancer and stereotactic body radiation therapy. Similar to the early adoption of IMRT radiotherapy, we postulate that there is a need to balance rapid adoption of PBT as an emerging technology and establishing randomized evidence upon which the recommendation for PBT can be made. The results of this study highlight the urgent need for comparative effectiveness PBT research in order to develop updated and high-quality evidence-based recommendations that can be used to inform its judicious use and to generate appropriate informational materials for patients.

## Additional files

Additional file 1: Appendix A: Data collection form. (DOCX 17 kb)

Additional file 2: Appendix B: Included and Excluded Proton Therapy Centers, sorted by country, state and city. (DOCX 19 kb)

Additional file 3: Appendix C: Proportion of All Disease Sites Mentioned on Proton Therapy Centre Websites. (DOCX 21 kb)

## Abbreviations

CI: Confidence Interval
CNS: Central Nervous System
DTCA: Direct-to-Consumer Advertising
FDA: Food and Drug Administration
IMRT: Intensity Modulated Radiation Therapy
OR: Odds Ratio
PBT: Proton Beam Therapy
PTC: Proton Therapy Centre
RT: Radiotherapy
