# Supplementary material for: Online advertising and marketing claims by providers of proton beam therapy: are they guideline-based?
Source: Radiat Oncol. 2018 Mar 15;13:43. doi: 10.1186/s13014-018-0988-z (PMC5856220; doi:10.1186/s13014-018-0988-z)
Supplement: Supplementary file 1 — Appendix A: Data collection form. (DOCX 17 kb) [file 13014_2018_988_MOESM1_ESM.docx]

Appendix A – Data Collection Form

| **Proton Website Direct to Consumer Advertising Data Collection Sheet** | | | | | | | |
| --- | --- | --- | --- | --- | --- | --- | --- |
| Collected by: | | | Date: | | | | |
| Name of Cancer Centre:  □ Academic (i.e. affiliated with a medical school) □ Community Hospital □ Private Practice | | | | | | | |
| NCCN Centre? □ Yes □ No | | | | | | | |
| What State(Country) is the Cancer Centre in? | | | | | | | |
| Population within 60mi: 180mi: | | | | | | | |
| Alexa Website Rank: | | | | | | | |
| Is there another Proton radiation unit in the same state? | | | | | □ Yes □ No | | |
| Is there another Proton radiation machine within 300km (180 mi)? | | | | | □ 1 □ ≥2 □ None | | |
| Does the Cancer Centre have a website? | | | | | □ Yes □ No | | |
| Is there a web page (or separate site) dedicated to Proton Radiation Therapy? | | | | | □ Page □ Site □ No | | |
| From the main cancer centre website (or proton dedicated website if applicable), how many clicks does it take to access webpage dedicated to Proton Radiation Therapy?  □ Directly on homepage/separate site □ One click □ Two clicks □ Three or more clicks | | | | | | | |
| **Manufacturer information involved in website?** | | | | | | | |
| Is there mention (or dedicated page) of brand/manufacturer of Proton RT machine? | | | | | | □ Yes □ No | |
| From the main cancer centre website (or proton dedicated website if applicable), how many clicks does it take to access webpage indicating the manufacturer of Proton RT machine?  □ Directly on homepage/separate site □ One click □ Two clicks □ Three or more clicks □ N/A | | | | | | | |
| Are claims of technologic superiority (i.e. more precise, more advanced, etc) made which are linked to the specific manufacturer of the Proton machine? (i.e. Machine X is the most advanced treatment machine available) | | | | | | □ Yes □ No □ N/A | |
| Is stock material from the manufacturer displayed on the website? (i.e. standard images available from the manufacturers website)  □ Stock image □ Stock text □ Stock video □ N/A | | | | | | | |
| If stock material is present from the manufacturer, is it acknowledged (by hyperlink or reference) that it comes from the manufacturer? | | | | | | □ Yes □ No □ N/A | |
| Is there a link to the manufacturer’s website present? | | | | | | □ Yes □ No □ N/A | |
| **Indications for Proton Radiation Therapy listed on website** | | | | | | | |
| Pediatric CNS | □ Yes □ No □ Added to Claim Appropriateness Review | | | | | | |
| Pediatric non-CNS (e.g. other solid tumours) | □ Yes □ No □ Added to Claim Appropriateness Review | | | | | | |
| Peds NOS |  | | | | | | |
| Adult CNS – Glioma (high/low grade) | □ Yes □ No □ Added to Claim Appropriateness Review | | | | | | |
| Adult CNS – Other primary brain | □ Yes □ No □ Added to Claim Appropriateness Review | | | | | | |
| Adult CNS – Eye/orbit | □ Yes □ No □ Added to Claim Appropriateness Review | | | | | | |
| Adult CNS – Spine | □ Yes □ No □ Added to Claim Appropriateness Review | | | | | | |
| Adult CNS – Benign (e.g. meningioma, AVM) | □ Yes □ No □ Added to Claim Appropriateness Review | | | | | | |
| CNS NOS |  | | | | | | |
| Esophagus | □ Yes □ No □ Added to Claim Appropriateness Review | | | | | | |
| Stomach | □ Yes □ No □ Added to Claim Appropriateness Review | | | | | | |
| Liver | □ Yes □ No □ Added to Claim Appropriateness Review | | | | | | |
| Colorectal | □ Yes □ No □ Added to Claim Appropriateness Review | | | | | | |
| Anal | □ Yes □ No □ Added to Claim Appropriateness Review | | | | | | |
| Bile Duct | □ Yes □ No □ Added to Claim Appropriateness Review | | | | | | |
| Pancreas | □ Yes □ No □ Added to Claim Appropriateness Review | | | | | | |
| GI NOS | □ Yes □ No □ Added to Claim Appropriateness Review | | | | | | |
| Head and Neck | □ Yes □ No □ Added to Claim Appropriateness Review | | | | | | |
| Lung | □ Yes □ No □ Added to Claim Appropriateness Review | | | | | | |
| Breast | □ Yes □ No □ Added to Claim Appropriateness Review | | | | | | |
| Lymphoma | □ Yes □ No □ Added to Claim Appropriateness Review | | | | | | |
| Sarcoma | □ Yes □ No □ Added to Claim Appropriateness Review | | | | | | |
| Prostate | □ Yes □ No □ Added to Claim Appropriateness Review | | | | | | |
| Bladder | □ Yes □ No □ Added to Claim Appropriateness Review | | | | | | |
| Kidney | □ Yes □ No □ Added to Claim Appropriateness Review | | | | | | |
| Testicular | □ Yes □ No □ Added to Claim Appropriateness Review | | | | | | |
| Recurrent Disease | □ Yes □ No □ Added to Claim Appropriateness Review | | | | | | |
| Bone | □ Yes □ No □ Added to Claim Appropriateness Review | | | | | | |
| Cervical | □ Yes □ No □ Added to Claim Appropriateness Review | | | | | | |
| Endometrial | □ Yes □ No □ Added to Claim Appropriateness Review | | | | | | |
| Ovarian | □ Yes □ No □ Added to Claim Appropriateness Review | | | | | | |
| Vaginal | □ Yes □ No □ Added to Claim Appropriateness Review | | | | | | |
| Vulvar | □ Yes □ No □ Added to Claim Appropriateness Review | | | | | | |
| Gyne NOS | □ Yes □ No □ Added to Claim Appropriateness Review | | | | | | |
| Skin | □ Yes □ No □ Added to Claim Appropriateness Review | | | | | | |
| Palliative Care | □ Yes □ No □ Added to Claim Appropriateness Review | | | | | | |
| Thymoma | □ Yes □ No □ Added to Claim Appropriateness Review | | | | | | |
| Other – Specify: | □ Yes □ No □ Added to Claim Appropriateness Review | | | | | | |
| **Does website make any claims (in general/listed for specific disease site) of:** | | | | | | | |
| **Increased efficacy** | | Proton Specific | | Generalized | | | None |
| Improved Survival/Cure/Disease Control | | □ | | □ | | | □ |
| Standard of Care | | □ | | □ | | | □ |
| **Decreased morbidity** | | Proton Specific | | Generalized | | | None |
| Fewer side effects/less toxic | | □ | | □ | | | □ |
| Quicker recovery time | | □ | | □ | | | □ |
| Are any side effects of therapy listed on website? | | □ | | □ | | | □ |
| Does website indicate increased quality of life? | | □ | | □ | | | □ |
| Dose Distribution Advantage (i.e. more precise) | | □ | | □ | | | □ |
| Comparison of Proton therapy to other radiation therapy techniques on website?  □ Increased efficacy □ Decreased morbidity □ No Comparison | | | | | | | |
| Comparison of Proton therapy to surgery on website?  □ Increased efficacy □ Decreased morbidity □ No Comparison | | | | | | | |
| Is evidence provided/referenced for claims made on the website?  □ Evidence provided for all claims (with reference)  □ Evidence provided any claim made on website (with reference)  □ Unreferenced claims of evidence (e.g. studies/research shows that…)  □ No evidence for any claims on website | | | | | | | |
| Does the website state it is a local/regional leader in administering:  □ Proton RT □ Radiation in general (no specific mention of Protons) □ No claim | | | | | | | |
| Does the website state that it is the only option in region for Proton Therapy? | | | | | | □ Yes □ No | |
| Is the length of time/patient volume they have been treating with Protons listed? | | | | | | □ Yes □ No | |
| Is there any discussion about the costs/insurance of Proton RT on the website? | | | | | | □ Yes □ No | |
| Are alternatives to Proton Therapy mentioned on their website? | | | | | | □ Yes □ No | |
| Is the use of Proton Therapy as a clinical trial mentioned on website? | | | | | | □ Yes □ No | |
| Are patient testimonials provided? □ Proton specific □ Any (non-proton) □ None | | | | | | | |
| Is the patient testimonial given in: □ Video format □ Text format □ None | | | | | | | |
| Is a patient testimonial being logged into the Excel file? | | | | | | □ Yes □ No | |
| Out of town patient section on website | | | | | | □ Yes □ No | |
