# Supplementary material for: Online advertising and marketing claims by providers of proton beam therapy: are they guideline-based?
Source: Radiat Oncol. 2018 Mar 15;13:43. doi: 10.1186/s13014-018-0988-z (PMC5856220; doi:10.1186/s13014-018-0988-z)
Supplement: Supplementary file 2 — Appendix B: Included and Excluded Proton Therapy Centers, sorted by country, state and city. (DOCX 19 kb) [file 13014_2018_988_MOESM2_ESM.docx]

| Appendix B –Included and Excluded Proton Therapy Centers, sorted by country, state and city. | | | |
| --- | --- | --- | --- |
| Included International Centres | | **Included North American Centres** | |
| Institution | **Location** | **Institution** | **Location** |
| Shanghai Proton and Heavy Ion Center | Shanghai, China | Mayo Clinic Proton Beam Therapy Program* | Phoenix, AZ, USA |
| Proton Therapy Centre | Prague, Czech Republic | James M Slater Proton Treatment & Research Center | Loma Linda, CA, USA |
| West German Proton Therapy Centre | Essen, Germany | Scripps Proton Therapy Center | San Diego, CA, USA |
| Heidelberg Ion-Beam Therapy Center | Heidelberg, Germany | Ackerman Cancer Center | Jacksonville, FL, USA |
| Rinecker Proton Therapy Center | Munich, Germany | University of Florida Health Proton Therapy Institute | Jacksonville, FL, USA |
| National Center of Oncological Hadrontherapy | Pavia, Italy | Marjorie and Leonard Williams Center for Proton Therapy | Orlando, FL, USA |
| Proton Beam Therapy Center | Hokkaido, Japan | Northwestern Medicine Chicago Proton Center | Chicago, IL, USA |
| Medipolis Proton Therapy and Research Center | Ibusuki, Japan | Willis-Knighton Health System | Shreveport, LA, USA |
| National Cancer Center Hospital East | Kashiwa, Japan | Maryland Proton Treatment Center | Baltimore, MD, USA |
| Southern TOHOKU Proton Therapy Center | Koriyama, Japan | Francis H Burr Proton Center at MGH | Boston, MA, USA |
| Aizawa Hospital Proton Therapy Center | Matsumoto, Japan | Mayo Clinic Proton Beam Therapy Program* | Rochester, MN, USA |
| Shizouka Cancer Center | Nagaizumi, Japan | S. Lee Kling Proton Therapy Center | St Louis, MO, USA |
| Nagoya Proton Therapy Center | Nagoya, Japan | Laurie Proton Therapy Center | New Brunswick, NJ, USA |
| Hyogo Ion Beam Medical Center | Shingu, Japan | ProCure Proton Therapy Center* | Somerset, NJ, USA |
| Proton Beam Therapy Center, University of Tsukuba | Tsukuba, Japan | Cincinnati Children's Proton Therapy Center | Cincinatti, OH, USA |
| Cyclotron Centre Bronowice | Krakow, Poland | University Hospitals Proton Therapy Center | Cleveland, OH, USA |
| Medico-Technical Complex | Dubna, Russia | ProCure Proton Therapy Center* | Oklahoma City, OK, USA |
| Petersburg Nuclear Physics Institute | St. Petersburg, Russia | Roberts Proton Therapy Center | Philadelphia, PA, USA |
| National Research Foundation iThemba LABS | Cape Town, South Africa | Provision Center for Proton Therapy | Knoxville, TN, USA |
| National Cancer Center Proton Therapy Center | Goyang, South Korea | St Jude Red Frog Events Proton Therapy Center | Memphis, TN, USA |
| Samsung Proton Therapy Center | Seoul, South Korea | MD Anderson Proton Therapy Centre | Houston, TX, USA |
| Skandion Clinic | Uppsala, Sweden | Texas Center for Proton Therapy | Irving, TX, USA |
| Paul Scherrer Institut Center for Proton Therapy | Villigen, Switzerland | Hampton University Proton Therapy Institute | Hampton, VA, USA |
| Chang Gung Memorial Hospital Proton Center | Taipei, Taiwan | SCCA Proton Therapy Center | Seattle, WA, USA |
|  | | | |
| Excluded International Centres | | **Excluded North American Centres** | |
| Institution and Reason for Exclusion | **Location** | **Institution and Reason for Exclusion** | **Location** |
| Wanjie Proton Therapy Centre (No English website) | Zibo, China | TRIUMF (Only treats eye/orbit) | Vancouver, BC, Canada |
| Clatterbridge Cancer Centre (Only treats eye/orbit) | Liverpool, England | UCSF Ocular Tumor Proton Program (Only treats eye/orbit) | San Francisco, CA, USA |
| Institut Mediterraneen de Proton Therapie (No English website) | Nice, France |  |  |
| Institut Curie Proton Therapy Centre (No English website) | Orsay, France |  |  |
| Helmholtz-Zentrum Berlin (No English website) | Berlin, Germany |  |  |
| Universitäts Protonen Therapie Dresden (No English website) | Dresden, Germany |  |  |
| Marburger Ionenstrahl-Therapiezentrum (No English website) | Marburg, Germany |  |  |
| CATANA Proton Therapy Facility (Only treats eye/orbit) | Catania, Italy |  |  |
| Azienda Provinciale per i Servizi Sanitari (No English website) | Trento, Italy |  |  |
| Fukui Proton Therapy Centre (No English website) | Fukui City, Japan |  |  |
| Tsuyama Chuo Hospital (No English website) | Okayama, Japan |  |  |
| Institute for Theoretical & Experimental Physics (No English website) | Moscow, Russia |  |  |
