# Supplementary material for: Online advertising and marketing claims by providers of proton beam therapy: are they guideline-based?
Source: Radiat Oncol. 2018 Mar 15;13:43. doi: 10.1186/s13014-018-0988-z (PMC5856220; doi:10.1186/s13014-018-0988-z)
Supplement: Supplementary file 3 — Appendix C: Proportion of All Disease Sites Mentioned on Proton Therapy Centre Websites. (DOCX 21 kb) [file 13014_2018_988_MOESM3_ESM.docx]

| **Appendix C** – Proportion of All Disease Sites Mentioned on Proton Therapy Centre Websites | | | | |
| --- | --- | --- | --- | --- |
| **Site** | **All Websites % (n)** (N = 46) | **US Websites % (n)**  (N = 22) | **International Websites % (n)**  (N = 24) | **US v. Int’l**  *p*-value |
| **Number of Sites Listed**  (mean ± SD) | 12.8 ± 6.1 | 15.5 ± 5.4 | 10.4 ± 5.8 | ***0.004*** |
| **Adult CNS (any)** | 89.1% (41) | 95.5% (21) | 83.3% (20) | 0.349 |
| Benign | 58.7% (27) | 63.6% (14) | 54.2% (13) | 0.515 |
| Glioma (High or Low Grade) | 45.7% (21) | 50.0% (11) | 41.7% (10) | 0.571 |
| Other (e.g. base of skull) | 65.2% (30) | 59.1% (13) | 70.8% (17) | 0.404 |
| Spine | 58.7% (26) | 77.3% (17) | 41.7% (10) | ***0.014*** |
| CNS NOS | 30.4% (14) | 45.5% (10) | 16.7% (4) | ***0.034*** |
| **Bone** | 6.5% (3) | 9.1% (2) | 4.2% (1) | 0.600 |
| **Breast** | 50.0% (23) | 77.3% (17) | 25.0% (6) | ***< 0.001*** |
| **Eye/Orbit** | 58.7% (27) | 68.2% (15) | 50.0% (12) | 0.211 |
| **GI (any)** | 76.1% (35) | 90.9% (20) | 62.5% (15) | ***0.024*** |
| Anal | 17.4% (8) | 36.4% (8) | 0% (0) | ***0.001*** |
| Colorectal | 39.1% (18) | 54.6% (12) | 25.0% (6) | ***0.040*** |
| Esophagus | 43.5% (20) | 54.6% (12) | 33.3% (8) | 0.147 |
| Liver | 56.5% (26) | 54.6% (12) | 58.3% (14) | 0.796 |
| Pancreatobiliary | 52.2% (24) | 68.2% (15) | 37.5% (9) | ***0.037*** |
| Stomach | 17.4% (8) | 31.8% (7) | 4.2% (1) | ***0.020*** |
| GI NOS | 6.5% (3) | 13.6% (3) | 0% (0) | 0.101 |
| **GU (any)** | 87.0% (40) | 95.5% (21) | 79.2% (19) | 0.190 |
| Bladder | 19.6% (9) | 27.3% (6) | 12.5% (3) | 0.276 |
| Kidney | 13.0% (6) | 4.6% (1) | 20.8% (5) | 0.190 |
| Prostate | 87.0% (40) | 95.5% (21) | 79.2% (19) | 0.190 |
| Testicular | 6.5% (3) | 9.1% (2) | 4.2% (1) | 0.600 |
| **Gynecologic (any)** | 30.4% (14) | 36.4% (8) | 25.0% (6) | 0.403 |
| Cervical | 19.6% (9) | 22.7% (5) | 16.7% (4) | 0.718 |
| Endometrial | 8.7% (4) | 9.1% (2) | 8.3% (2) | 1.00 |
| Ovarian | 2.2% (1) | 4.6% (1) | 0% (0) | 0.478 |
| Vaginal | 6.5% (3) | 9.1% (2) | 4.2% (1) | 0.600 |
| Vulvar | 4.4% (2) | 9.1% (2) | 0% (0) | 0.223 |
| Gyne NOS | 8.7% (4) | 18.2% (4) | 0% (0) | ***0.045*** |
| **Head and Neck** | 87.0% (40) | 90.9% (20) | 83.3% (20) | 0.667 |
| **Lymphoma** | 41.3% (19) | 68.2% (15) | 16.7% (4) | ***< 0.001*** |
| **Lung** | 76.1% (35) | 95.5% (21) | 58.3% (14) | ***0.003*** |
| **Palliative** | 2.2% (1) | 4.6% (1) | 0% (0) | 0.478 |
| **Pediatric Tumours (Any)** | 82.6% (38) | 95.5% (21) | 70.8% (17) | ***0.049*** |
| Pediatric CNS | 47.8% (22) | 59.1% (13) | 37.5% (9) | 0.143 |
| Pediatric non-CNS | 47.8% (22) | 59.1% (13) | 37.5% (9) | 0.143 |
| Pediatric NOS | 39.1% (18) | 45.5% (10) | 33.3% (8) | 0.400 |
| **Recurrent Disease** | 54.4% (25) | 54.6% (12) | 54.2% (13) | 0.979 |
| **Sarcoma** | 67.4% (31) | 81.8% (18) | 54.2% (13) | ***0.046*** |
| **Skin** | 8.7% (4) | 4.6% (1) | 12.5% (3) | 0.609 |
| **Thymoma** | 6.5% (3) | 4.6% (1) | 8.3% (2) | 0.405 |

**CNS** – Central nervous system; **NOS** – Not otherwise specified; **GI** – Gastrointestinal; **GU** – Genitourinary
